# Efficacy of Accelerated Intermittent Theta Burst Stimulation (aiTBS) for Depression: A Systematic Review and Meta-Analysis of Randomized Controlled Trials

**DOI:** 10.1192/j.eurpsy.2026.10247

**Published:** 2026-07-31

**Authors:** B. R. Yamamoto, J. M. Santos, A. R. Brunoni

**Affiliations:** Universidade de São Paulo, São Paulo, Brazil

## Abstract

**Introduction:**

Theta burst stimulation (TBS) is a more time-efficient alternative to repetitive transcranial magnetic stimulation (rTMS) for depression, especially in resistant cases. Conventional protocols, however, require weeks of daily visits, limiting adherence. Accelerated intermittent TBS (aiTBS, ≥2 sessions/day) seeks to shorten treatment and improve outcomes, but evidence remains limited, warranting systematic evaluation of its efficacy, safety, and clinical utility.

**Objectives:**

To assess the efficacy of aTBS for major depressive disorder (MDD) and bipolar depression (BD); to compare the effectiveness of hyper-accelerated versus moderately accelerated schedules; to investigate potential sources of heterogeneity from subgroup analyses, including targeting method (e.g. neuronavigated approaches), acceleration schedule and type of TBS protocol.

**Methods:**

This Systematic Review and meta-analysis followed PRISMA guidelines and was registered on PROSPERO (CRD420251081143). PubMed, Embase, Cochrane CENTRAL, ClinicalTrials.gov, and MedRxiv were searched up to July 2025. Eligibility criteria included randomized controlled trials (RCTs) enrolling adults (18–65 years) with MDD or BD treated with any aTBS protocol versus sham. The primary outcome was depressive symptom reduction; secondary outcomes were response and remission rates. Random-effects pairwise meta-analyses were performed; risk of bias was assessed with Cochrane RoB-2.

**Results:**

Thirteen sham-controlled RCTs (n=488) were included. Compared with sham, aiTBS significantly reduced depressive symptoms (Hedges’ g = -0.60; 95% CI [-0.96, -0.24]; p=0.0011), a medium-to-large beneficial effect according to Cohen’s benchmarks. Heterogeneity was moderate (I²=64.1%, 95% CI [35.0–80.2]) (figure 1); leave-one-out analyses confirmed robustness. No evidence of publication bias was detected (Egger’s p=0.66). Response rates (10 RCTs, n=373) (figure 2) favored aiTBS (RR = 2.00; 95% CI [1.39, 2.89]; p=0.0002) with low heterogeneity (I²=28.5%). Remission rates (9 RCTs, n=360) (figure 3) were nearly doubled (RR = 1.97; 95% CI [1.00, 3.86]; p=0.049), with low-to-moderate heterogeneity (I²=34.5%). Subgroup analyses suggested stronger effects with functional connectivity-based targeting, higher stimulation doses (≥50 sessions, ≥90,000 pulses), 10 sessions/day, and 50-minute intersession intervals.

**Image 1:**
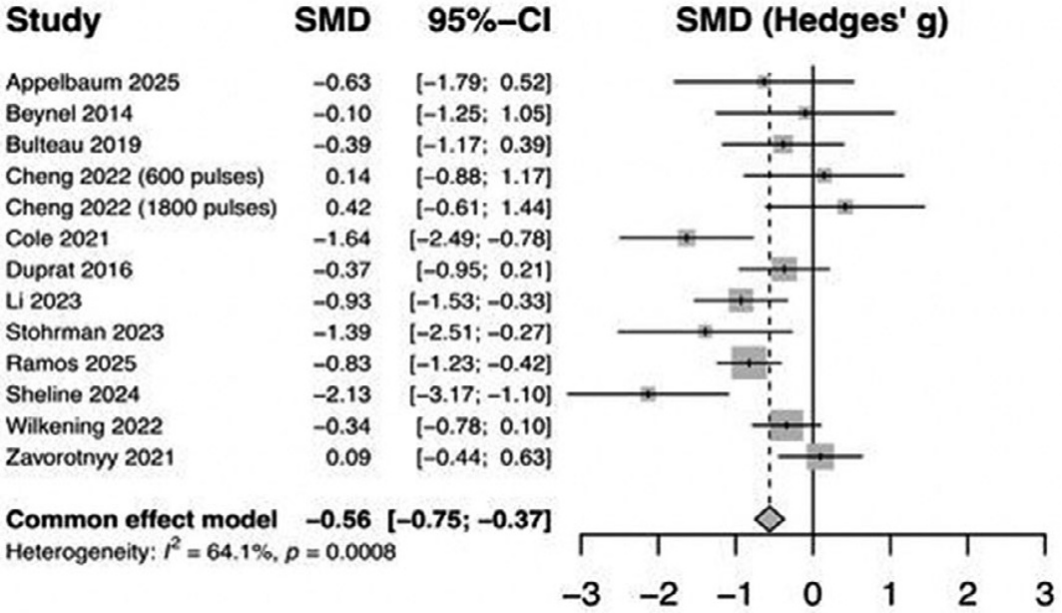
Image 1: Long description.

**Image 2:**
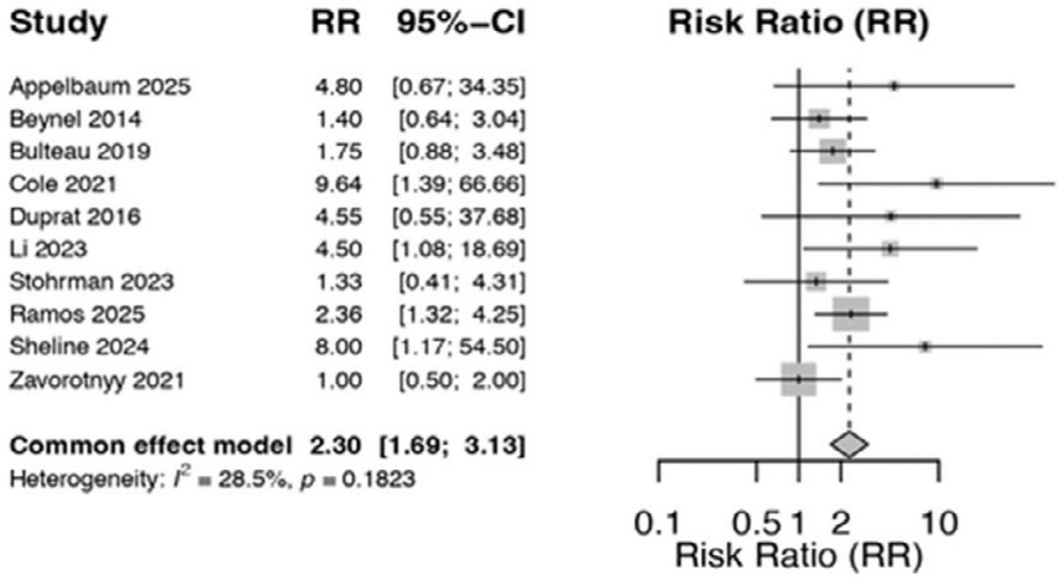
Image 2: Long description.

**Image 3:**
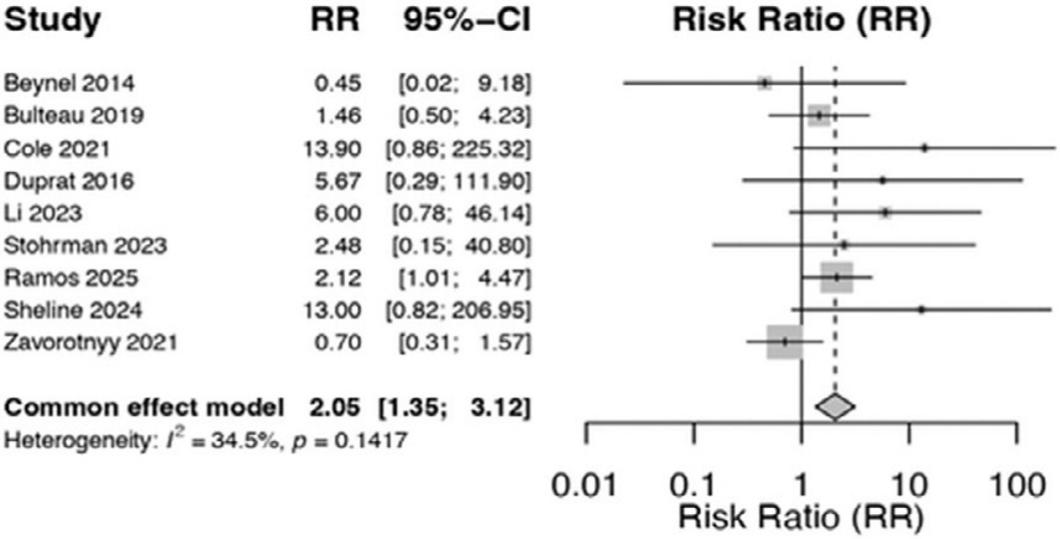
Image 3: Long description.

**Conclusions:**

aiTBS appears to be an effective and well-tolerated intervention for depression, providing significant reductions in symptoms and doubling response and remission rates. Efficacy is influenced by stimulation dose and target localization. These findings support aTBS as a rapid-acting, safe, and clinically relevant neuromodulation strategy, with potential to enhance adherence and accessibility in psychiatric care.

**Disclosure of Interest:**

None Declared

## Long descriptions

### Image 1: Long description

A forest plot with columns for Study, S M D (Standardized Mean Difference), 95% C I (Confidence Interval), and a visual plot of S M D Hedges’ g. The x-axis of the plot ranges from -3 to 3 with a vertical solid line at 0 (no effect) and a dashed vertical line at the aggregate mean of -0.56.

Individual study data from top to bottom:

* Appelbaum 2025: -0.63 [-1.79; 0.52]

* Beynel 2014: -0.10 [-1.25; 1.05]

* Bulteau 2019: -0.39 [-1.17; 0.39]

* Cheng 2022 (600 pulses): 0.14 [-0.88; 1.17]

* Cheng 2022 (1800 pulses): 0.42 [-0.61; 1.44]

* Cole 2021: -1.64 [-2.49; -0.78]

* Duprat 2016: -0.37 [-0.95; 0.21]

* Li 2023: -0.93 [-1.53; -0.33]

* Stohrman 2023: -1.39 [-2.51; -0.27]

* Ramos 2025: -0.83 [-1.23; -0.42]

* Sheline 2024: -2.13 [-3.17; -1.10]

* Wilkening 2022: -0.34 [-0.78; 0.10]

* Zavorotnyy 2021: 0.09 [-0.44; 0.63]

At the bottom, the Common effect model shows an S M D of -0.56 with a 95% C I of [-0.75; -0.37], represented by a grey diamond centered on the dashed line. Heterogeneity is reported as I super 2 = 64.1%, p = 0.0008.

Navigate back to Image 1:.

### Image 2: Long description

The forest plot consists of a table on the left and a graphical representation on the right. The x-axis of the plot is labeled Risk Ratio R R on a logarithmic scale with markers at 0.1, 0.5, 1, 2, and 10. A solid vertical line represents the null effect at R R equals 1.0, and a dashed vertical line represents the common effect model at 2.30.

Individual study data from top to bottom:

* Appelbaum 2025: R R 4.80, C I 0.67 to 34.35.

* Beynel 2014: R R 1.40, C I 0.64 to 3.04.

* Bulteau 2019: R R 1.75, C I 0.88 to 3.48.

* Cole 2021: R R 9.64, C I 1.39 to 66.66.

* Duprat 2016: R R 4.55, C I 0.55 to 37.68.

* Li 2023: R R 4.50, C I 1.08 to 18.69.

* Stohrman 2023: R R 1.33, C I 0.41 to 4.31.

* Ramos 2025: R R 2.36, C I 1.32 to 4.25.

* Sheline 2024: R R 8.00, C I 1.17 to 54.50.

* Zavorotnyy 2021: R R 1.00, C I 0.50 to 2.00.

At the bottom, the Common effect model shows an R R of 2.30 with a 95 percent C I of 1.69 to 3.13, represented by a diamond shape centered on the dashed line. Heterogeneity is reported as I super 2 equals 28.5 percent, p equals 0.1823.

Navigate back to Image 2:.

### Image 3: Long description

A forest plot table with four columns. The first column lists nine studies. The second column lists R R values. The third column lists 95% C I. The fourth column is a graphical Risk Ratio R R plot on a logarithmic scale from 0.01 to 100.

Data rows from top to bottom

* Beynel 2014. R R 0.45. C I 0.02 to 9.18.

* Bulteau 2019. R R 1.46. C I 0.50 to 4.23.

* Cole 2021. R R 13.90. C I 0.86 to 225.32.

* Duprat 2016. R R 5.67. C I 0.29 to 111.90.

* Li 2023. R R 6.00. C I 0.78 to 46.14.

* Stohrman 2023. R R 2.48. C I 0.15 to 40.80.

* Ramos 2025. R R 2.12. C I 1.01 to 4.47.

* Sheline 2024. R R 13.00. C I 0.82 to 206.95.

* Zavorotnyy 2021. R R 0.70. C I 0.31 to 1.57.

Summary statistics at the bottom

* Common effect model. R R 2.05. C I 1.35 to 3.12. Represented by a diamond shape centered at 2.05 on the plot.

* Heterogeneity. I super 2 equals 34.5%. p equals 0.1417.

The plot includes a vertical solid line at R R equals 1 indicating no effect and a vertical dashed line aligned with the common effect model result.

Navigate back to Image 3:.

